# Bufalin Inhibits Cytokine Storm by Regulating TLR4/TLR3 Signaling Pathway

**DOI:** 10.1002/iid3.70318

**Published:** 2026-02-04

**Authors:** Xixi Liu, Chencheng Li, Jing Yang, Weiguang Zhang, Zhongxiao Hu, Xiaoli Zhang, Reaila Jianati, Fang Tian, Xingbin Dai, Zuqiong Xu, Biqing Chen, Xuejun Zhu

**Affiliations:** ^1^ Affiliated Hospital of Nanjing University of Chinese Medicine First Clinical Medical College of Nanjing University of Chinese Medicine Nanjing China; ^2^ Department of General Medicine, The People's Hospital of Lianxi District Jiujiang City Jiujiang China; ^3^ Wisdom Lake Academy of Pharmacy Xi'an Jiaotong‐Liverpool University Suzhou China; ^4^ Lianshui Hospital of Traditional Chinese Medicine Huaian China

**Keywords:** bufalin, cytokine storm, inflammation, TOLL‐like receptors

## Abstract

**Background:**

Bufalin is one main component of the dried venom from *Bufo gargarizans Cantor*, which has anti‐tumor, cardiotonic, anti‐inflammatory and other physiological activities. However, in recent years, researchers have mainly paid attention to its anti‐tumor effect and neglected its anti‐inflammatory effect.

**Methods:**

We used lipopolysaccharide (TLR4 ligand) and poly inosinic acid (TLR3 ligand) to stimulate cultured macrophages to induce inflammatory condition. Transcriptome sequencing and molecular experiments were performed to investigate the underlying mechanism.

**Results:**

Bufalin could significantly reduce the production of pro‐inflammatory factors (IL‐6, TNF‐α, IL‐1β, IL‐8, CXCL10, etc.), through inhibiting the phosphorylation of IKBα and IRF3, and thus down‐regulating Toll‐like receptor pathway. Molecular docking predicted that one of the molecular targets of bufalin is MD‐2 coupled with lipopolysaccharide‐activated TLR4.

**Conclusion:**

These findings not only support the pharmacological basis of using toad to treat inflammatory diseases in the Chinese medical history, but also provide a promising anti‐inflammatory drug candidate for future clinical application.

## 1 Introduction

Inflammation is a defensive response of the immune system to harmful stimuli, which is helpful to remove invasive pathogens, promote tissue repair and maintain internal homeostasis [1, 2]. However, excessive or persistent inflammatory reaction can also cause acute injury or some chronic diseases. Cytokine storm is an uncontrolled systemic inflammatory reaction caused by excessive cytokines, which can lead to multiple organ failure and even death. Cytokine storms have existed in many infectious diseases, including severe acute respiratory syndrome and COVID‐19 [3], as well as in immunotherapy such as chimeric antigen receptor T cell therapy. Monocytes/macrophages are the key mediators of cytokine storm [4, 5]. Excessive macrophage reaction could be harmful to the host [6]. It is supposed that inflammation could be mitigated by inhibiting and reducing M1 macrophages and/or activating and increasing M2 macrophages. [7].

Inflammation is related to the development and deterioration of most cancers [8]. Many anti‐tumor targets are also anti‐inflammatory targets, and many anti‐tumor drugs have anti‐inflammatory capability as well [9, 10]. As a widely used Chinese medicine famous for its anti‐tumor effect [11], the ethanol extract of the venom from *Bufo gargarizans Cantor* has also been used to treat various inflammatory diseases including tonsillitis and sore throat [12, 13] in the history. In the current decades, it has been used to treat hepatitis B and some purulent infections in clinic, and is believed to have anti‐inflammatory and anti‐microbial effects [14]. It mechanism has been revealed to reduce the infiltration of activated F4/80+ and/or CD68+cells, significantly decreasing M1 macrophages and proinflammatory cytokine expression [15]. Bufalin is one of the main effective components of the venom from *Bufo gargarizans Cantor*, with relatively low side effects [16]. It has a variety of physiological effects including stimulating myocardial contraction and anti‐tumor activity [14, 15].

In recent years, with the in‐depth study of signaling pathways, the molecular mechanisms underlying the anti‐inflammatory effects of bufalin have become increasingly clear, confirming its role as a veritable “multi‐target” anti‐inflammatory agent.

### 1.1 Impact on the Toll‐Like Receptor (TLR) Signaling Pathway

The TLR signaling pathway plays a crucial role in innate immune responses by recognizing pathogen‐associated molecular patterns and damage‐associated molecular patterns, thereby triggering inflammatory responses. Multiple experimental studies have shown that bufalin can inhibit TLR‐triggered inflammatory responses. Toll‐interacting protein is an intracellular adapter protein that negatively regulates TLR signaling through its interaction with TLRs, thereby controlling the intensity and duration of the inflammatory response [17].

### 1.2 Impact on the Nuclear Factor‐Kappa B (NF‐κB) Pathway

NF‐κB transcription factors play a central role in regulating inflammatory responses, immune responses, cell survival, and apoptosis. Studies have demonstrated that bufalin inhibits the activation of NF‐κB, which is typically induced by inflammatory stimuli such as cytokines, PAMPs, and oxidative stress. By suppressing NF‐κB activation, bufalin effectively blocks the transcription of pro‐inflammatory genes, thereby reducing the production of inflammatory mediators [18].

### 1.3 Impact on the Mitogen‐Activated Protein Kinase (MAPK) Pathway

The MAPK signaling pathway(including ERK, JNK, and p38 MAPK) plays an important role in inflammatory responses by regulating inflammatory gene expression, cytokine production, cell proliferation, and apoptosis [19]. Research indicates that bufalin can inhibit the phosphorylation and activation of MAPKs, including ERK, JNK, and p38. By blocking the activation of these kinases, bufalin attenuates downstream signaling events, thereby inhibiting the production of pro‐inflammatory mediators [20].

### 1.4 Impact on the Janus Kinase (JAK) Signaling Pathway

The JAK‐STAT signaling pathway is a crucial signal transduction system that regulates inflammatory responses, immune responses, cell proliferation, differentiation, and death. Dysregulation of this pathway is closely associated with various inflammatory diseases. Studies have confirmed that bufalin effectively inhibits JAK kinase activation and STAT protein phosphorylation, thereby reducing STAT transcriptional activity and the production of downstream inflammatory mediators. Through the regulation of the JAK‐STAT signaling pathway, this compound achieves dual suppression of both the production and activation of inflammatory mediators [21].

Bufalin is transforming from a traditional “cardiac glycoside toxin” into a rising star in modern drug development for inflammatory diseases, thanks to its well‐defined and potent multi‐target anti‐inflammatory mechanisms. For patients who respond poorly to existing anti‐inflammatory drugs (e.g., corticosteroids, biologics), who are prone to drug resistance, or who experience significant side effects (e.g., those with advanced cancer‐related inflammation or autoimmune diseases), bufalin, with its unique multi‐target mechanism of action, holds promise as an alternative or adjunctive therapy to break through current treatment bottlenecks, demonstrating high clinical translational value.

This study evaluated the effect of bufalin on cytokine storm at the cellular level, proposed a possible targeting molecular mechanism by analyzing transcriptome sequencing data and molecular docking.

## 2 Materials and Methods

### 2.1 Isolation and Extraction of Human Peripheral Blood Mononuclear Cell

Peripheral blood samples were collected from six healthy volunteers, with the blood collection process approved by the Ethics Committee of Jiangsu Provincial Hospital of Traditional Chinese Medicine (Approval number 2022NL‐124‐02). The blood samples were added with ficoll separation (Tianjin Haoyang Biological Products Technology Co. Ltd., China) in a ratio of 1:1 and centrifuged at 750 *g* for 20 min to purify peripheral blood mononuclear cells (PBMCs). Extracted PBMCs were then cultured in RPMI 1640 medium (Gibco Company, United States) containing 10% fetal bovine serum (Zhejiang Tianhang Biotechnology Co. Ltd., China) in a 5%CO_2_ incubator at 37°C.

### 2.2 Cell Culture

Two monocytic cell lines, THP‐1 and U937 (ATCC, USA), were cultured in RPMI 1640 medium containing 10% fetal bovine serum at 37°C and 5%CO_2_. The cells were stimulated with 100 ng/mL phorbol 12‐myristate 13‐acetate(PMA) (MCE, United States) for 24 h to induce differentiation into M0 macrophages.

### 2.3 Cellular Inflammatory State Simulation

PBMCs and PMA‐induced monocytic cells were stimulated by 500 ng/mL lipopolysaccharide (LPS) (Sigma, Germany) or 50 ug/mL Poly inosinic acid (Poly I: C) (InvivoGen, United States) for 24 h to simulate an inflammatory state for cultured cells. Bufalin (MCE) was co‐cultured with the inflammatory cells for 24 h as the experimental group. The blank group was only medium without drugs.

### 2.4 Giemsa Staining

Drip Giemsa staining solution A on the smear containing THP‐1 cells, and let the staining solution cover the whole specimen smear for staining. Color for 1 min; Drop Giemsa dyeing solution B on solution A, so that solution A and solution B are fully mixed and dyed for 4–10 times. Minutes; Washing, drying and microscopic examination.

### 2.5 Detection of Macrophage Phenotype by Flow Cytometry

The cultured cells were washed with PBS, digested and centrifuged with 5 mM EDTA, with cell density adjusted to 1 × 10^6^ cells/mL. The cells were incubated with fluorescent‐labeled antibodies CD11b‐APC (D12, BD Biosciences, United States) and CD86‐PE (IT2.2, Biolegend, United States) independently at 4°C for 30 min and then went through flow cytometry.

### 2.6 Detection of Inflammatory Cytokines by Cytometric Bead Array (CBA) and Enzyme‐Linked Immunosorbent Assay (ELISA)

Cultured medium was collected and centrifuged at 1500 *g* for 3 min. The supernatant was collected for subsequent cytometric bead array with the Human Twelve Cytokines Detection Kit (Qingdao Riskell Biotechnology Co. Ltd., China) and ELISA (Ruixin Biotechnology Co. Ltd., China). CXCL10 and IFN‐β were measured by ELISA, while all the other cytokines were measured by CBA. All steps in this experiment were carried out according to the manufacturer's instruction.

### 2.7 Transcriptome Sequencing

1 × 10^6^ cells per sample were collected by centrifuge and lysed byTrizol. Total RNA was extracted and its quality was assessed by QUBIT and agarose gel electrophoresis. cDNA libraries are constructed by enriching for mRNA with Oligo(dT). The cDNA library was then sequenced on the Illumina platform to generate 6 G data. The sequencing data were analyzed to quantify gene expressions. Gene set enrichment analysis (GSEA) and over representation analysis (ORT) were conducted by R package ClusterProfiler.

### 2.8 Real‐Time Quantitative Polymerase Chain Reaction (qPCR)

1 × 10^6^ cells per sample were collected by centrifuge at 1500 g for 3 min. Total RNA was extracted by RNA extraction kit (Shanghai Feijie Biotechnology Co. Ltd., China) and reverse transcripted (Applied Biological Materials Inc, Canada) into cDNA according to the instructions, and qPCR was performed with qPCR mix (Applied Biological Materials Inc, Canada) according to the following procedure: 40 cycles of 95°C for 15 s, and 60°C for 1 min.

### 2.9 RNA Interference

Cultured monocytes were stimulated with 100 ng/mL PMA for 24 h and then 50 ug/mL Poly I:C for 24 h to simulate an inflammatory state. siRNAs targeting TLR4 and TRIF (synthesized by Shanghai Shenggong Bioengineering, China) were transfected respectively with Lipofectamine 2000 (Thermo Fisher Scientific, United States) for 24 h, while negative control siRNA was used as the control. The culture medium was refreshed after 6 h.

### 2.10 Western Blot Analysis

Cultured cells were lysed by lysis buffer with protease and phosphatase inhibitors, and centrifuged at 14,000 g for 10 min to extract proteins. Proteins were quantified using the bicinchoninic acid assay kit and separated by10% sodium dodecyl sulfate‐polyacrylamide gel electrophoresis (10 μg of each sample) and transferred onto polyvinylidene difluoride (PVDF) membranes for 90 min at 100 V. The membranes were incubated with 5% skim milk for 30 min for blocking, followed by incubation withprimary antibodies against TLR3 (1:1000, Polyclonal, Affinity, United States), TLR4 (1:1000, 3G9A4, Proteintech, China), phospho‐IκBα (1:1000, 14D4, Cell Signaling Technology, United States), IκBα (1:1000, L35A5, Cell Signaling Technology), GAPDH (1E6D9,1:50000, Proteintech), IRF3 (1:5000, 1E6G8, Proteintech), and phospho‐IRF3 (1:1000, Polyclonal, Cell Signaling Technology) at 4°C overnight and secondary antibodies goat anti‐rabbit IgG (1:5000, Polyclonal, Proteintech) and goat anti‐mouse IgG (1:5000, Polyclonal, Proteintech) at room temperature for 1 h. The blots were finally exposed using the ECL chemicalluminescence method. Images were analyzed by Image J software.

### 2.11 Molecular Docking

The structures of MD‐2protein (PDB ID:2E59) and bufalin were prepared by protein Preparation Guide and LigPrep module, respectively. Using SP precision in GLIDE to flexibly connect through standard protocols [22, 23]. Ten conformations were generated and scored by glide score. The best scoring conformation is used for structural analysis, and the graph is generated by Pymol.

### 2.12 Statistical Analysis

Statistical analyses were performed by SPSS17.0. R Studio and GraphPad were adopted to draw figures. Student's *t* test was used to compare means between two groups if normal distribution was conformed. Statistically significance was set at *p* < 0.05.

## 3 Results

### 3.1 Bufalin Inhibits LPS‐Induced Macrophage Activation In Vitro

**Figure 1:**
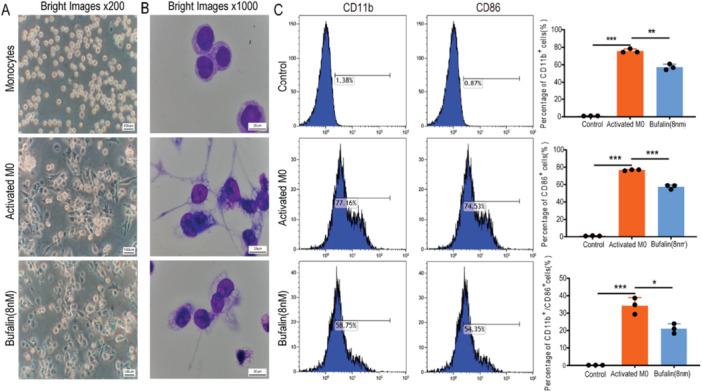
Bufalin inhibits PMA and LPS‐induced macrophage activation. (A) Effects of Bufalin treatment on the morphology of macrophages under phase contrast microscope. (B) Under the white field microscope after Giemsa staining, the morphology of macrophages before and after Bufalin treatment was affected. (C) Flow cytometry showed the phenotype and activation level of macrophages. Control were THP‐1 cells, Activates M0 were treated with PMA (100 ng/mL) for 24 h, and then LPS (500 ng/mL) was added for 24 h. Bufalin was treated with PMA + LPS and then treated with Bufalin 8 nM for 24 h. The data is expressed as the average of three replicates. *p < 0.05, **p < 0.01, *** *p* < 0.001.

Under normal conditions, human monocytic THP‐1 cells were round with smooth edges and no pseudopodia (Figures 1A and 1B, top). After stimulation with PMA and LPS for 24 h, the cells displayed characteristics of activated macrophages, namely, irregular morphology, and spindle‐shaped pseudopodia (Figures 1A and 1B, middle). Treatment with bufalin (8 nM) returned them to unactivated morphology of macrophages, such as reducing the number of pseudopodium (Figures 1A and 1B, bottom). Flow cytometry analysis confirmed that THP‐1 cells were activated to M1 type macrophages (CD11b^+^CD86^+^) after PMA and LPS stimulation, while bufalin treatment led to a decrease in the percentage of M1 type macrophages (Figure 1C).

### 3.2 Bufalin Inhibits Cytokine Storm in LPS‐Activated Macrophages

**Figure 2:**
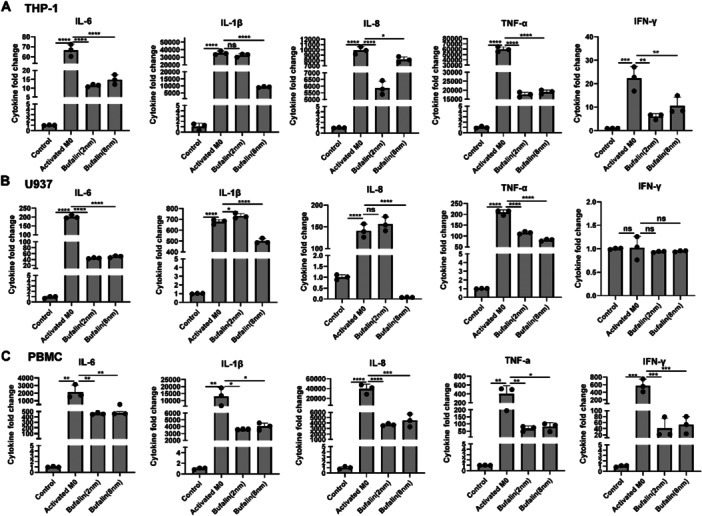
Bufalin inhibits inflammatory factors secreted by LPS‐induced activated macrophages. (A) the effect of bufalin on inflammatory factors secreted by monocytes/macrophages (THP‐1) induced by PMA and LPS. (B) Effects of Bufalin on inflammatory factors secreted by monocytes/macrophages (U937) induced by PMA and LPS. (C) Effect of Bufalin on inflammatory factors secreted by peripheral blood mononuclear cells (PBMC) stimulated by LPS. A control is THP‐1 cells, B control is U937 cells, C control is PBMC, Activates M0 were treated with PMA (100 ng/mL) for 24 h, and then LPS (500 ng/mL) was added for 24 h. Bufalin was treated with PMA + LPS and then treated with Bufalin 8 nM for 24 h. The data is expressed as the average of three independent replicates. *p < 0.05, **p < 0.01 and ***p < 0.001. ns, non‐significance.

Activated macrophages secreted a large number of inflammatory cytokines (IL‐6, IL‐1β, IL‐8, TNF‐α, etc.). These cytokine levels could be significantly mitigated by bufalin treatment (Figure 2A). This anti‐inflammatory effect of bufalin has been repeated in human monocytic cell line U937 and human primary PBMC (Figures 2B and 2C).

### 3.3 Bufalin Mitigates Cytokine Storm in LPS‐Induced Macrophages Through Toll‐Like Receptor Signaling Pathway

**Figure 3:**
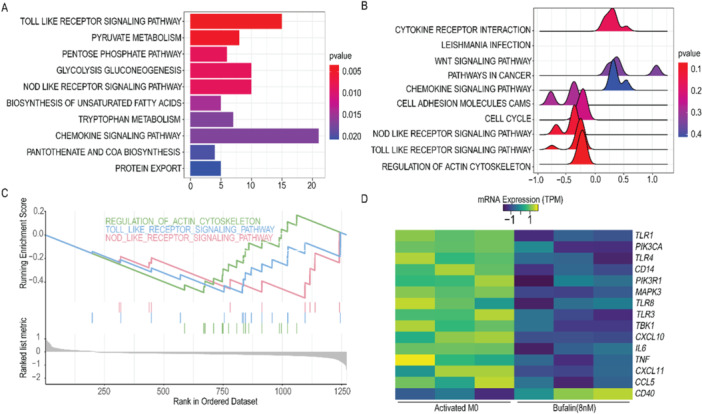
Bufalin down‐regulates Toll‐like receptor signaling pathway in activated macrophages. (A) signal pathways of genes with significant change times were analyzed by ORT enrichment(*p* < 0.05), and the horizontal axis was the number of gene changes of activated monocytes/macrophages before and after Bufalin treatment. (B) Significantly changed genes enriched in 10 pathways by GSEA analysis (*p* value is not filtered). The horizontal axis is gene expression ratio of activated monocytes/macrophages before and after cinobufotalin treatment, taking as log10, color of the ridges indicate the significance level. (C) GSEA and ORT enrichment analysis show the enrichment trend of overlapping significant change signal pathways, with the vertical axis indicating the enrichment score and the horizontal axis indicating the accumulated gene number. (D) Heatmap shows the expression changes of TOLL pathway related genes in activated macrophages before and after Bufalin treatment. Control is an activated macrophage. Activated M0 were treated with PMA (100 ng/mL) for 24 h and then LPS (500 ng/mL) was added for 24 h. Bufalin was treated with PMA + LPS and then treated with Bufalin 8 nM for 24 h.

To explore the molecular mechanism underlying the inflammation inhibitory effect of bufalin, gene expression profiles of the activated and inactivated macrophages before and after bufalin treatment were compared. Over representation test (ORT) analysis revealed ten pathways significantly enriched in bufalin‐treated macrophages (*p *< 0.05) (Figure 3A). Gene set enrichment analysis (GSEA) also found ten pathways significantly changed(*p* value is not filtered) (Figure 3B). GSEA and ORT analyses overlapped on three pathways, which were all significantly down‐regulated by bufalin treatment (Figure 3C). Among them, TOLL‐like receptor pathway has been reported to be associated with inflammation [24]. Most genes in the TOLL‐like receptor pathway were significantly down‐regulated (Figure 3D).

### 3.4 Bufalin Abates Cytokine Storm in Both LPS and Poly I: C Activated Macrophages Through the TLR3 Pathway

**Figure 4:**
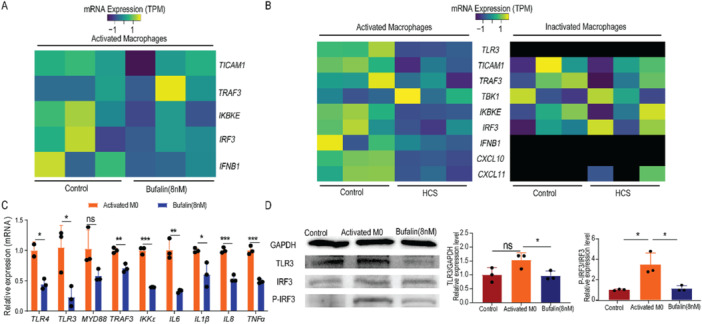
Bufalin down‐regulates TLR4 and TLR3 signaling pathways in activated macrophages. (A) thermogram shows the expression changes of TLR3 pathway related genes of activated monocytes/macrophages before and after Bufalin treatment. (B) thermogram shows the mRNA expression changes of TLR3 pathway related genes of resting and activated monocytes/macrophages before and after cinobufagin treatment. Black blocks indicate no mRNA detected by sequencing. (C) Bufalin on gene expression in TLR4/TLR3 pathway of activated macrophages. (D) Western blotting showed the effect of Bufalin on the expression of TLR3, IRF3, P‐IRF3 proteins in activated macrophages, and the grey analysis of western blotting blot. Inactivated Macrophsges were THP‐1 cells, and Activated M0/Activated Macrophsges were THP‐1 cells, were treated with PMA(100 ng/mL) for 24 h, and then LPS (500 ng/mL) was added for 24 h. Bufalin 8 nM is PMA + LPS, and then Bufalin 8 nM is added for 24 h. The data is expressed as the average of three independent replicates. *p < 0.05, **p < 0.01, ***p < 0.001. ns, non‐significance.

Further analysis of the RNAseq data revealed that bufalin significantly down‐regulated TLR3‐TRAF3‐IKKε‐ pIRF3 pathway in the LPS‐activated macrophages (Figure 4A). Treatment of the ethanol extract of Chinese Toad Venom, which is the raw extract where bufalin is one major component, resulted in a similar expression pattern, i.e., down‐regulation of the TLR3 pathway in the LPS‐activated macrophages(Figure 4B). However, the TLR3 pathway was not significantly affected when the monocytes were not activated (Figure 4B). The significant down‐regulation of TLR3, TRAF3 and IKKε were validated by qPCR (Figure 4C), and western blotting confirmed reduction of the downstream IRF3 phosphorylation (Figure 4D).

**Figure 5:**
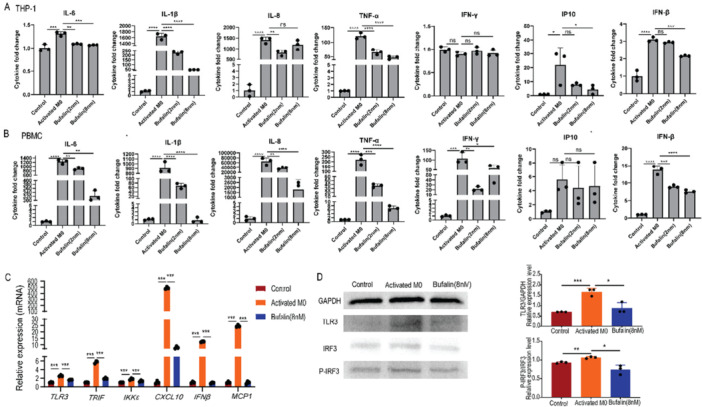
Bufalin inhibits the inflammatory response induced by Poly I:C via the regulation of the TLR3/P‐IRF3 signaling pathway. (A) The effects of Bufalin on inflammatory factors secreted by activated monocytes/macrophages (THP‐1). (B) The effects of Bufalin on inflammatory factors secreted by peripheral blood mononuclear cells (PBMC) after stimulation. (C) The effects of Bufalin on gene expression in the TLR3 signaling pathway of macrophages after Poly I:C stimulation. (D) Western blotting showing the expression of TLR3, IRF3, and P‐IRF3 in activated macrophages and Bufalin‐treated cells, as well as the gray analysis of the Western blotting bands. A/C/D Control represents THP‐1 cells, and B Control represents primary human PBMC cells. Activated M0 refers to the treatment of PMA (100 ng/mL) for 24 h followed by the addition of Poly I:C (50 ng/mL) for 24 h. Bufalin 8 nM refers to the addition of Bufalin 8 nM after PMA + Poly I:C treatment for 24 h. The data is expressed as the average of three independent replicates. *p < 0.05, **p < 0.01, ***p < 0.001. ns, non‐significance.

Since one important pathogenic ligand of TLR3 is viral double‐stranded RNA (dsRNA), we then investigated whether direct activation of TLR3 by Poly I: C, a synthetic analog of dsRNA, could be impeded by bufalin [25, 26]. Poly I: C stimulated macrophages to produce a large amount of cytokines (IL‐1β, IL‐8, TNF‐α, CXCL10, IFN‐β, etc.), while bufalin treatment could significantly mitigate them (Figure 5A). The results were replicated with human PBMC (Figure 5B). It was further confirmed that bufalin could down‐regulate the TLR3 pathway, including TLR3, TRIF, and IKKε, in Poly I: C‐activated macrophages as well (Figure 5C), and then decreased the phosphorylation of IRF3 (Figure 5D), which led to downstream decrease of cytokines such as CXCL10 (encoding IP‐10).

### 3.5 TLR4 Plays a Necessary Role in the Effect of Bufalin on Inflammation‐Activated Macrophages

**Figure 6:**
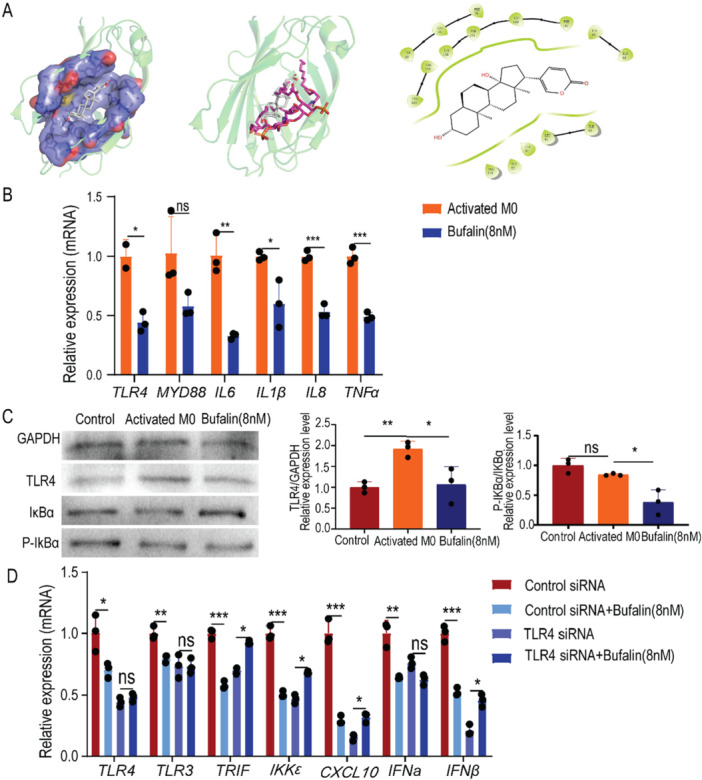
Bufalin indirectly downregulates the TLR3 pathway by affecting TLR4. (A) Bufalin binds to TLR4‐MD2. (B) Bufalin on gene expression in TLR4 pathway of activated macrophages. (C) Western blotting showing the expression of TLR4, IκBα, and P‐IκBα in activated macrophages and Bufalin‐treated cells, as well as the gray analysis of the Western blotting bands. (D) Expression of related genes in the TLR3 pathway after TLR4 siRNA transfection and Bufalin treatment. Control siRNA/TLR4 siRNA was stimulated with PMA (100 ng/ml) for 24 h, followed by the addition of Poly I:C (50ug/ml) for 24 h, and Control siRNA/TLR4 siRNA treatment for 6 h followed by the addition of medium for 18 h. Control siRNA +Bufalin/TLR4 siRNA+ Bufalin was stimulated with PMA (100 ng/ml) for 24 h, followed by the addition of Poly I:C (50ug/ml) for 24 h, and Control siRNA/TLR4 siRNA treatment for 6 hoursfollowed by the addition of Bufalin 8 nM for 18 h. The data is expressed as the average of three independent replicates, * means that compared with the Control siRNA group, *p < 0.05, **p < 0.01, *** *p* < 0.001. ns, non‐significance.

Since the origin of TLR3 pathway, TLR3, was down‐regulated as well, it was supposed that there was at least a molecular target other than TLR3 in the TOLL‐like receptor pathway, which would be direct target of bufalin. Thus, we predicted targets of bufalin by the SuperPRED software. TLR4, in the LPS‐activated state binding with myeloid differentiation protein 2 (MD2), hit with a high score as a potential target of bufalin (Table 1). Molecular docking of bufalin and LPS‐activated TLR4‐MD2 complex displayed relatively high affinity with a docking pocket on MD2, composed of 133 CYS‐136 VAL, 147 PHE‐149 LEU‐151 PHE‐153 ILE, 44 ILE‐46 ILE, and 61 LEU‐63 ILE (Figure 6A).

**Table 1:** Prediction of bufalin molecular targets.

Target name	Gene	Probability	Model accuracy
LSD1/CoREST complex	KDM1A	95%	97%
DNA‐ (apurinic or apyrimidinic site) lyase	APEX1	94%	91%
Nuclear factor NF‐kappa‐B p105 subunit	NFKB1	92%	96%
Dual specificity protein kinase CLK4	CLK4	92%	94%
Transcription intermediary factor 1‐alpha	TRIM24	91.1%	96%
Mineralocorticoid receptor	NR3C2	91%	100%
NT‐3 growth factor receptor	NTRK3	88%	96%
Acetyl‐CoA carboxylase 1	ACACA	87.2%	93%
Casein kinase II alpha/beta	CSNK2B	85%	99%
Cyclooxygenase‐1	PTGS1	83%	90%
Glycine receptor subunit alpha‐1	GLRA1	81%	91%
Proteasome component C5	PSMB1	81.1%	90%
Methionine aminopeptidase 2	METAP2	79%	97%
Indoleamine 2,3‐dioxygenase	IDO1	79%	96%
Dual specificity protein phosphatase 3	DUSP3	78%	94%
Lysosomal Pro‐X carboxypeptidase	PRCP	77%	100%
Cathepsin D	CTSD	75%	99%
Histone deacetylase 8	HDAC8	75%	94%
Androgen receptor	AR	74%	96%
ADAM10	ADAM10	73%	97.5%
Stimulator of interferon genes protein	STING1	72%	95%
Cyclin‐dependent kinase 2/cyclin E1	CCNE1	71%	93%
Tyrosine‐protein kinase ITK/TSK	ITK	71%	95%
Toll‐like receptor 4	TLR4	71%	92.5%
Voltage‐gated N‐type calcium channel alpha‐1B subunit	CACNA1B	70%	97%
C5a anaphylatoxin chemotactic receptor	C5AR1	70%	93%
Lipoxin A4 receptor	FPR2	70%	100%

Since bufalin was predicted to bind LPS‐activated TLR4 protein complex, we reexamined whether the downstream pathway of TLR4 was responsive to bufalin treatment. The results found that some cytokines including IL‐6 and TNF‐α which were at the downstream of TLR4 pathway were significantly decreased (Figure 6B). Western Blot further confirmed inhibited phosphorylation by bufalin of IκBα, which was elevated in activated macrophages (Figure 6C). In order to find out whether TLR4 is necessary for bufalin's anti‐inflammatory effect on monocyte‐derived macrophages, we further interfered TLR4 with small interfer RNA. Bufalin significantly downregulated the TLR4/TLR3 pathway in the control group, but when TLR4 was knocked down, this inhibitory effect disappeared (Figure 6D). It suggests that TLR4 is necessary for the anti‐inflammatory effect of bufalin.

## 4 Discussion

**Figure 7:**
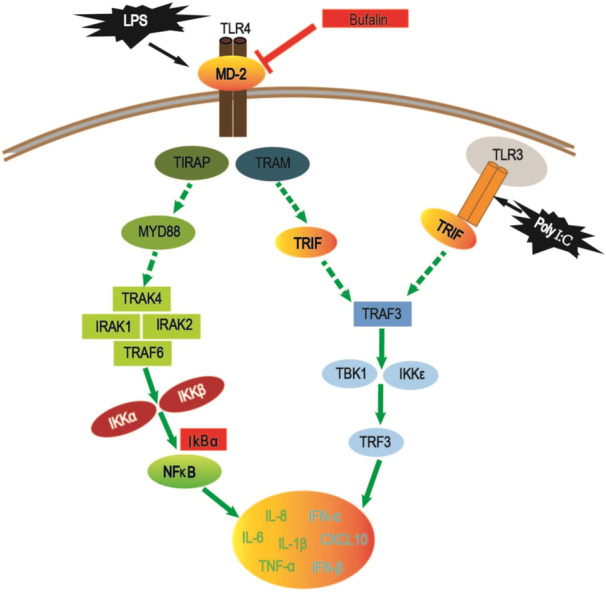
Illustration showing the potential mechanism of bufalin on LPS/Poly I:C activated macrophages.

Our previous study has found that Chinese Toad extract could inhibit monocytic inflammation through TLR4 pathway [27]. This study found that bufalin, as one of the main components of Chinese Toad extract, could inhibit inflammatory reaction induced by bacterial membrane component LPS or virus RNA analog Poly I:C at the cellular level, and its mechanism might be through direct binding to TLR4‐MD2 complex, inhibiting complex aggregation, down regulating TLR4/P‐IκBα and TLR3/P‐IRF3 pathways (Figure 7).

It is considered that the over‐activation of the innate immunity system is one major factor for COVID‐19 induced cytokine storm [28]. Large‐scale single cell RNA sequencing revealed that macrophage was the main source of inflammatory cytokines and the main host cell of SARS‐CoV‐2 virus in COVID‐19 patients [29]. Therefore, discovering efficient inhibitor on uncontrolled macrophage activation could be helpful for many diseases involving over‐inflammation. Current known macrophage inhibitors include BTK inhibitor GDC‐0853 which prevents macrophage polarization by inhibiting TLR4/NF‐κB signaling pathway [30], dexamethasone which inhibits activity of M1 macrophage [31], and cholesterol synthesis inhibitor statins which promote the formation of M2 macrophages [32, 33]. However, most treatments have obvious side effects [34]. As an active compound extracted from natural product, bufalin has shown a more prominent anti‐inflammatory activity with fewer side effects, indicating greater research and application prospect.

Toll‐like receptors constitute a key signal system that regulates macrophage function. Different toll‐like receptors recognize distinct pathogen‐related molecular patterns and trigger inflammation by producing pro‐inflammatory cytokines [24]. TLR4 recognizes bacterial LPS and produces inflammatory cytokines such as TNF‐α, IL‐1β, and IL‐6 [35], while TLR3 recognizes viral double‐stranded RNA and induces inflammatory chemokines/cytokines including CXCL10 and IFN‐β [25, 36]. TLR3 helps to activate NFκB and IRF3 in macrophages [37]. By inhibiting TLR3/NLPR3/NFκB/IRF3 signaling pathway [38], airway inflammation could be improved [39]. As bufalin can inhibit TLR3‐IRF3 pathway, it suggests that bufalin may be used to relieve inflammation caused by virus infection. It is generally believed that as family members, TLR4 and TLR3 function in parallel. However, the current study found that TLR3 pathway was affected when LPS stimulated TLR4. Other research has also reported indirect effect of TLR4 on TLR3 pathway [40, 41]. Tian et al. proved that LPS induces TLR3 expression through the TLR4‐MyD88‐IRAK‐TRAF6‐NF‐κB signaling pathway in monocytes [41], which was replicated in the current study. Furthermore, bufalin could inhibit the pro‐inflammatory effect of LPS. When TLR4 was knocked down, the genes involved in TLR3 pathway were significantly down‐regulated with downstream cytokines decreased. Moreover, after TLR4 knockdown, bufalin's inhibitory effect on TLR3 pathway and cytokine levels diminished. As suggested by molecular docking, bufalin might indirectly inhibit TLR3 pathway by acting on TLR4‐MD2 complex. MD2 is a secreted glycoprotein which will bind the extracellular domain of TLR4 upon LPS stimulation, and triggers a series of downstream inflammatory reactions. Antibiotics which reduce its binding with LPS lead to down‐regulation of inflammation [42]. Therefore, bufalin might competitively target TLR4‐MD2 complex, which mitigates cytokine storm in macrophages by directly down‐regulating TLR4‐pIκBα pathway and indirectly down‐regulating TLR3‐pIRF3 pathway (Figure 7).

However, M1 and M2 macrophages are not the only cell types involved in excessive release of cytokines. The effect of bufalin on other cytokine‐releasing cells has not been touched in this study. Whether TLR4‐MD2 complex is the direct target of bufalin needs further experiments. In addition, animal model of cytokine storm has not been tested in this study, which is still far from clinical guidance. Besides, bufalin is a cardiac glycoside with low water solubility and slow clearance in vivo. The general therapeutic dose is about 60% of the toxic dose, thus, the therapeutic window is narrow. Therefore, it needs to be modified before clinical application. At present, there are some modified molecules, such as BF211, which need further experiments to investigate their influence on cytokine storm and its related diseases.

To summary, this study shows that bufalin inhibits cytokine production in macrophages stimulated by bacteria and virus induced inflammation, and the potential underlying molecular mechanism is to regulate TLR4‐pIκBα pathway directly and TLR3‐pIRF3 pathway indirectly. This study provides a new insight for developing effective drugs to rapidly alleviate diseases involving macrophagic cytokine storm, such as COVID‐19 [28, 29], by inhibiting over‐activated monocytes.

## 5 Conclusion

As the central instigator of the cytokine storm, the dysregulated activation of macrophages is a critical factor in many severe infectious diseases (such as COVID‐19) and complications of immunotherapy. Consequently, inhibiting overactivated macrophages represents a promising strategy for overcoming the cytokine storm. Bufalin, which can suppress the secretion of various inflammatory factors, including IL‐6 and IP‐10, from macrophages, is a highly promising candidate drug for treating cytokine storms. It may provide a novel and effective therapeutic approach for managing severe diseases associated with cytokine storms, including COVID‐19, CAR‐T therapy‐induced cytokine release syndrome (CRS), and hemophagocytic lymphohistiocytosis (HLH). This initiative represents a novel exploration into utilizing traditional Chinese medicine and its active compounds to treat severe cytokine storm‐related diseases.

## Author Contributions

**Xixi Liu:** conceptualization, data curation, formal analysis, writing – original draft. **Chencheng Li:** conceptualization, data curation, formal analysis, writing – original draft. **Jing Yang:** data curation, investigation. **Weiguang Zhang:** data curation, investigation. **Zhongxiao Hu:** data curation, investigation. **Xiaoli Zhang:** data curation, investigation. **Reaila Jianati:** data curation, investigation. **Fang Tian:** Resources. **Xingbin Dai:** resources. **Zuqiong Xu:** resources. **Biqing Chen:** conceptualization, formal analysis, funding acquisition, project administration, supervision validation, writing – review and editing. **Xuejun Zhu:** conceptualization, formal analysis, funding acquisition, project administration, supervision validation, writing – review and editing.

## Ethics Statement

The authors are accountable for all aspects of the work in ensuring that questions related to the accuracy or integrity of any part of the work are appropriately investigated and resolved. All procedures performed in studies involving human participants were in accordance with the ethical standards of the institutional and national research committee and with the Helsinki Declaration (as revised in 2013). And written informed consent was obtained from the patient donor in this study. This study was approved by the Ethical Review Committee Affiliated Hospital of Nanjing University of Chinese Medicine/Jiangsu Province Hospital of Chinese Medicine (Approval number 2022NL‐124‐02).

## Conflicts of Interest

The authors declare no conflicts of interest.

## Data Availability

The datasets used and/or analyzed during the current study are available from the corresponding authors on reasonable request.
